# Combined Administration of Curcumin and Chondroitin Sulfate Alleviates Cartilage Injury and Inflammation *via* NF-κB Pathway in Knee Osteoarthritis Rats

**DOI:** 10.3389/fphar.2022.882304

**Published:** 2022-05-19

**Authors:** Ting Guan, Liu-Gang Ding, Bao-Yuan Lu, Jia-Yi Guo, Mei-Yin Wu, Zhi-Qun Tan, Shao-Zhen Hou

**Affiliations:** ^1^ School of Pharmaceutical Sciences, Guangzhou University of Chinese Medicine, Guangzhou, China; ^2^ School of Food Science and Engineering, South China University of Technology, Guangzhou, China; ^3^ Infinitus (China) Company Ltd., Guangzhou, China; ^4^ Guangzhou Hongyun Medical Scientific and Technological Co., Ltd., Guangzhou, China; ^5^ Institute for Memory Impairments and Neurological Disorder, University of California, Irvine, Irvine, CA, United States

**Keywords:** curcumin, chondroitin sulfate (CS), knee osteoarthritis (KOA), inflammation, NF-κB

## Abstract

**Objective:** Osteoarthritis (OA) is a degenerative chronic disease that most often occurs in the knee joint. Studies have shown that some food supplements, such as curcumin and chondroitin sulfate, are effective in treating knee osteoarthritis (KOA) by exhibiting different protective effects. In this study, we further investigated the combined therapeutic effects of curcumin and chondroitin sulfate on cartilage injury in rats with arthritis.

**Methods:** An experimental KOA model was induced by monosodium iodoacetate (MIA) in rats. All rats were randomly divided into five groups: Ctrl (control), model (saline), Cur (20 mg/kg curcumin in saline), CS (100 mg/kg chondroitin sulfate in saline), and CA (20 mg/kg curcumin and 100 mg/kg chondroitin sulfate in saline); drugs were given 2 weeks after MIA injection. The histomorphological changes of cartilage were observed by safranin fast green staining, H&E staining, and micro-CT scanning. Also, the levels of PGE2, TNF-α and IL-1β in the arthral fluid and serum were determined by the ELISA kits. The activities of SOD, CAT, COMP, MMP-3, and type II collagen were detected by biochemical kits. The expressions of TLR4, p-NF-κB, NF-κB, and COX-2 in cartilage were detected by Western blot.

**Results:** Data show that serum levels of IL-1β (*p* < 0.05), SOD (*p* < 0.0001), and MMP-3 (*p* < 0.001) were downregulated significantly in the CA group when compared to those in the model group. Meanwhile, obvious repair of cartilage with higher contains collagen II (*p* < 0.0001) could be observed in the CA group than the ones in Cur or CS group. In addition, significant downregulation of the expression of p-p65/p65 (*p* < 0.05) was found in the CA group.

**Conclusion:** Our findings showed that combined administration of curcumin and chondroitin sulfate could exert better repair for KOA in rat models. This may hold great promise for discovering potential drugs to treat KOA and may improve treatment options for it.

## Introduction

Knee osteoarthritis (KOA) is a kind of degenerative bone and joint disease with knee swelling, pain, and limited movement as the main clinical manifestations, and it is the major disability disease among middle-aged and elderly people (Vaishya et al., 2016). OA is a metabolically active and dynamic process in which various biochemical and mechanical factors combine to cause destruction (Xia et al., 2014). KOA has been widely recognized as an immune-related disease, and as such inflammation and oxidative stress are frequently discussed in the pathogenesis and development of KOA (Hunter and Bierma-Zeinstra, 2019). Oxidative stress may contribute to OA, which is characterized by elevated levels of reactive oxygen species (ROS). It increased oxidative activity and mediates the action of many pro-inflammatory cytokines, such as tumor necrosis factor-α (TNF-α), and interleukin 1β (IL-1β) (Altay et al., 2015; Malek et al., 2015). Chondrocytes secrete pro-inflammatory cytokines in a stressful environment, and these cytokines induce the synthesis of matrix metalloproteinases (MMPs), inflammatory cytokines, and chemokines. In addition, pro-inflammatory cytokines reduce the synthesis of collagen and proteoglycans and activate the production of various inflammatory mediators and proteases (Goldring and Otero, 2011; Lee et al., 2014; Hunt et al., 2020).

The NF-κB signaling pathway plays an important role in regulating the expression of pro-inflammatory factors, adhesion molecules, and chemokines, as well as playing a central role in inflammation, cell proliferation, differentiation, and apoptosis. Because NF-κB is involved in many biological processes, abnormalities of the NF-κB pathway are common. It can be observed in many diseases, such as arthritis, cancer, and autoimmune diseases (Kumar et al., 2004; Courtois and Gilmore, 2006; Herrington et al., 2016; Choi et al., 2019). As NF-κB dimers bind to IκB protein, once IκB protein is degraded, the NF-κB signaling pathway is freely activated, damaging chondrocytes and leading to the development of KOA (Lepetsos et al., 2019). NF-κB induces catabolic gene expression through NF-κB response elements located in promoters of MMP1, MMP9, MMP3, and ADAMTS5 genes, as well as expression of major pro-inflammatory and destructive mediators that promote OA, including cyclooxygenase 2 (cox-2), prostaglandin E2 (PGE2), and inducible nitric oxide synthase (iNOS) (Choi et al., 2019). Therefore, the TLR4/NF-κB signaling pathway has a high value in the study of KOA therapeutic mechanisms.

In recent years, a great deal of progress has been made in research on the treatment of KOA and promising herbal formulations and ingredients have been discovered that have the ability to slow down the progression of KOA, promote cartilage repair, and improve knee joint disease (Wang et al., 2010). Many clinical trials on curcumin supplementation have been conducted on various autoimmune diseases including osteoarthritis (Yang et al., 2019), and curcumin can reduce inflammation in knee osteoarthritis rats by blocking TLR4/MyD88/NF-κB signal pathway (Zhang and Zeng, 2019). Chondroitin sulfate is a basic component of cartilage and synovial fluid, which increases type II collagen to stimulate the anabolic process of cartilage metabolism. Loss of chondroitin sulfate from the cartilage would lead to OA (Bishnoi et al., 2016). Chondroitin sulfate or chondroitin sulfate has been recommended as a therapeutic intervention in OA (Kubovy et al., 2012; Schneider et al., 2012).

Therefore, we hypothesized that the combined administration of curcumin and chondroitin sulfate has a better protective effect on KOA rats. In this experiment, we explored the cooperative effect of curcumin and chondroitin sulfate in MIA-induced knee osteoarthritis in rats. It was found that co-administration of curcumin and chondroitin sulfate was more effective on reducing knee swelling and inflammation, promoting chondrocyte proliferation, and joint repair in rats with knee osteoarthritis.

In recent years, due to aging, obesity, and traumatic knee injuries, osteoarthritis (OA) has become numerous and widespread and a disabling disease of the elderly worldwide (Hunter and Bierma-Zeinstra, 2019). In a population-based cohort study, the lifetime risk of symptoms of knee osteoarthritis was 45% (Woolf and Pfleger, 2003). The treatment of OA is still dominated by nonsteroidal anti-inflammatories; however, the long-term use of nonsteroidal anti-inflammatories usually causes severe gastrointestinal side effects. Therefore, disease-modifying KOA drugs or food supplements are urgently needed to reduce pain and improve function while delaying the structural progression of the disease (Murphy et al., 2008).

## Methods

### Materials

A food supplement of curcumin was provided by Chr. Hansen Holding A/S (Batch: PO100790, Hoers Holm, Denmark). Food supplement of chondroitin sulfate (Chondroitin sulfate Na, 38.80%, derived from a novel enzymatic hydrolyzed chicken sternal cartilage extract) was obtained from Meitek Technology Co., Ltd (CJ19101003, Qingdao, China). MIA was purchased from Sigma-Aldrich (St. Louis, MO, United States); EDTA decalcification solution (quick decalcification) was purchased from Servicebio (Wuhan, China); enzyme-linked immunosorbent assay (ELISA) kits of TNF-α, IL-1β, SOD, CAT, MMP-3, COL-II, PGE2, and COMP were obtained from Jiangsu Meimian Industrial Co., Ltd. (Nanjing, China). NF-kappaB and p-NF-kappaB antibodies were purchased from Cell Signaling Technology (MA, United States). cox-2, TLR4, and GAPDH antibodies were purchased from Signalway Antibody Co., Ltd. (MD, United States). Goat anti-mouse IgG (H + L) HRP and goat anti-rabbit IgG (H + L) HRP were purchased from Affinity Biosciences (OH, United States).

**Table:** 

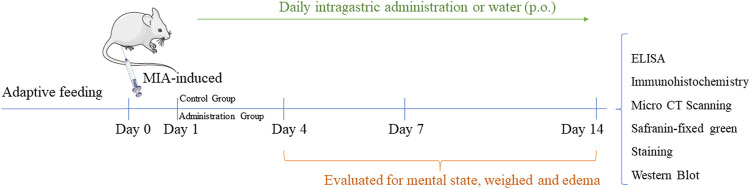

### Animals

A total of 50 male SPF SD rats (SCXK 2019-0047) of 2 months old, weighing 220–250 g, were obtained from the Experimental Animal Center of Guangzhou University of Chinese Medicine (License Number: SCXK 2019-0202). The animals were housed in a room with the controlled temperature (22 ± 2°C) and humidity (55 ± 10%) and light–dark cycles of 12 h.

### Induction of KOA

The rats were anesthetized with 0.4% pentobarbital sodium (i.p.). After anesthesia, the leg was flexed keeping the right knee at 90° angle; the needle was positioned through the infrapatellar ligament, and the MIA solution was injected into the intra-articular region of the right knee. The animals of the control group received an intra-articular injection of sterile saline.

### Experimental Design

KOA animals were divided into four groups, model group (*n* = 10) administrated with saline orally, Cur group (*n* = 10) received an oral dose of 20 mg/kg curcumin supplement (Nakagawa et al., 2020), CS group (*n* = 10) treated with chondroitin sulfate (CS) at a dose of 100 mg/kg, and CA group (*n* = 10) co-given with 20 mg/kg curcumin supplement and 100 mg/kg chondroitin sulfate. All the animals were treated with saline, curcumin supplement (Cur), or/and chondroitin sulfate (CS) for 2 weeks after modeling. All procedures were performed according to the Laboratory Animal Science guidelines. All the animals were weighed and evaluated for mental state and edema before KOA induction and at days 1, 4, 7, and 14 after the saline or MIA injection (Udo et al., 2016).

On the fifteenth day, the animals from each group were euthanized, and the samples of blood serum, intra-articular lavage of knees were collected and stored at −80°C for further analysis. The knee joints of the normal control group (*n* = 5), the MIA-induced group (*n* = 5), and other experiment groups (*n* = 5) were removed and stored in 4% formalin for histological evaluation.

Normal saline of 50 μl was injected into the joint cavity using an insulin syringe, which was mixed with synovial fluid by moving the tibia of the rats. Finally, 50 μl of normal saline was injected to fill the joint cavity, and the rats were forced to bend their knees to compress the joint cavity, while the joint fluid was sucked out. The joint cavity fluid is used to test ELISA kits.

### Evaluation of Articular Edema

From day 1 until day 14 after the start of arthritis, clinical severity was graded semi-quantitively on a scale of 0–2 for each hind paw, according to changes in redness and swelling: 0: no changes; 0.5: slight; 1.0: moderate; 1.5: marked; 2.0: maximal swelling and redness. The Vernier caliper was also used to measure the transversal diameter of the knee to confirm the knee swelling (Yamada et al., 2019).

### Mankin Scores

After 14 days from the onset of arthritis, the rats were euthanized, knee joints were isolated, and processed for histological examination. The dissected joints and treated samples were evaluated using Mankin scores based on the following Table 1 (van der Sluijs et al., 1992; Gerwin et al., 2010).

**TABLE 1:** Mankin Score—scoring standard of articular pathology (Supplementary Material).

Classification	Score
Structure	
Normal	0
Surface damage	1
Pannus and surface damage	2
Shallow fissures formed reach moving layers	3
Locally reaching the fracture of the bone radiation layer	4
Reach the weight-bearing area of cartilage defect in the calcified layer of deep bone	5
Full-thickness cartilage defect	6
Cell	
Normal	0
Excessive and disordered cells	1
Cell clustering	2
Less cells	3
Safranin fast green staining normal	
Normal	0
Mild dyeing	1
Medium dyeing	2
Deep dyeing	3
Loss of dyeing	4
Integrity of tidal line	
Complete	0
Incomplete	1
Incomplete blood vessels pass through	2
Aggregate score	**0–14**

### CT Scanning

Micro-CT was used to scan and determine the bone tissue. After the animals were killed, the right joint was isolated, fixed with 4% paraformaldehyde, stored at room temperature, and soaked with normal saline for 1 day after 2 days. The femur was dried and put into the specimen cup vertically and fixed securely using a circular foam column. After the samples were fixed, the scanning voltage was set to 80 kV, and the scanning current was set to 100 μA. The distal femur was scanned along the long axis of the femur with a scanning thickness of 15 μm, a filter of AL0.5 mm, and a resolution of 1,000 × 666 to obtain continuous plane micro-CT images.

### Histological Evaluation

The right knees of the rats were removed and fixed in formaldehyde. This study focused on the distal articular cartilage of the femur (right knee). The knees were decalcified in 0.5 mol/L EDTA for up to 20 days. After 24 h of fixation, the rat tibia was placed in EDTA decalcification solution and shaken in a 37°C shaker for 24 h a week, and the decalcification solution was changed once a week and the process lasted for 3 weeks. During the process, the entire joint needs to be taken out, and a needle is used to test whether the bone can be pierced. When the bone becomes soft and rubbery, the decalcification is successful.

Afterward, the knees were cut on a sagittal plane, for later use in routinely processed for histopathology and stained with hematoxylin and eosin, also with safranin fast green staining.

### ELISA

Serum levels of TNF-α (Lot: MM-0180R1) and IL-1β (Lot: MM-0047R1) at 7 and 14 days were detected using the enzyme-linked immunosorbent assay (ELISA) kits. The detection method was performed according to the instructions of the manufacturers.

The activities of SOD (Lot: MM-0386R1), CAT (Lot: MM-20447R1), COMP (Lot: MM-20306R1), PGE2 (Lot: MM-0068R1), MMP-3 (Lot: MM-0112R1), and collagen type II (Lot: MM-70372R1) were determined using the appropriate test kit according to the manufacturer’s instructions (ELISA kits were obtained from Jiangsu Meimian Industrial Co., Ltd. (Nanjing, China)). All of them were measured using a multimode microplate reader (Multiskan GO1510; Thermo Fisher Scientific, Vantaa, Finland).

### Western Blot Assay

The method for Western blot assay was performed as described in the previous study (Chen et al., 2020). Briefly, the cartilage tissues were lysed in RIPA buffer with protease inhibitor and phosphatase inhibitor. Cartilage tissues (50 mg) were separated by 8%–12% sodium dodecyl sulfate-polyacrylamide gel electrophoresis and transferred onto nitrocellulose membranes and then incubated with antibodies (NF-kappaB (Catalog No. 8242S, CST, Danvers, MA, United States), p-NF-kappaB (Catalog No. 3033S, CST, Danvers, MA, United States), cox-2 (Catalog No. 12282S, SAB, China), TLR4 (Catalog No. 35463, SAB, China), GAPDH (Catalog No. 41549-2, SAB, China). Then, after incubation with the secondary antibody at room temperature, the ECL kit showed protein bands. Finally, the bands were quantified by ImageJ software (National Institutes of Health, United States).

### Statistical Analysis

We used GraphPad Prism version 8 (GraphPad Software, Inc., San Diego, CA, United States) for statistical analysis. The results were evaluated statistically using a one-way analysis of variance (ANOVA) followed by post hoc Tukey’s test or Student’s t-test when appropriate. The data were expressed as means ± standard deviation (SD) for each group. The values presenting *p <* 0.05 were considered significant.

## Results

### Combined Administration of Curcumin and Chondroitin Sulfate (CA) Inhibited Redness and Swelling of Joints in MIA-Induced Rats

**FIGURE 1:**
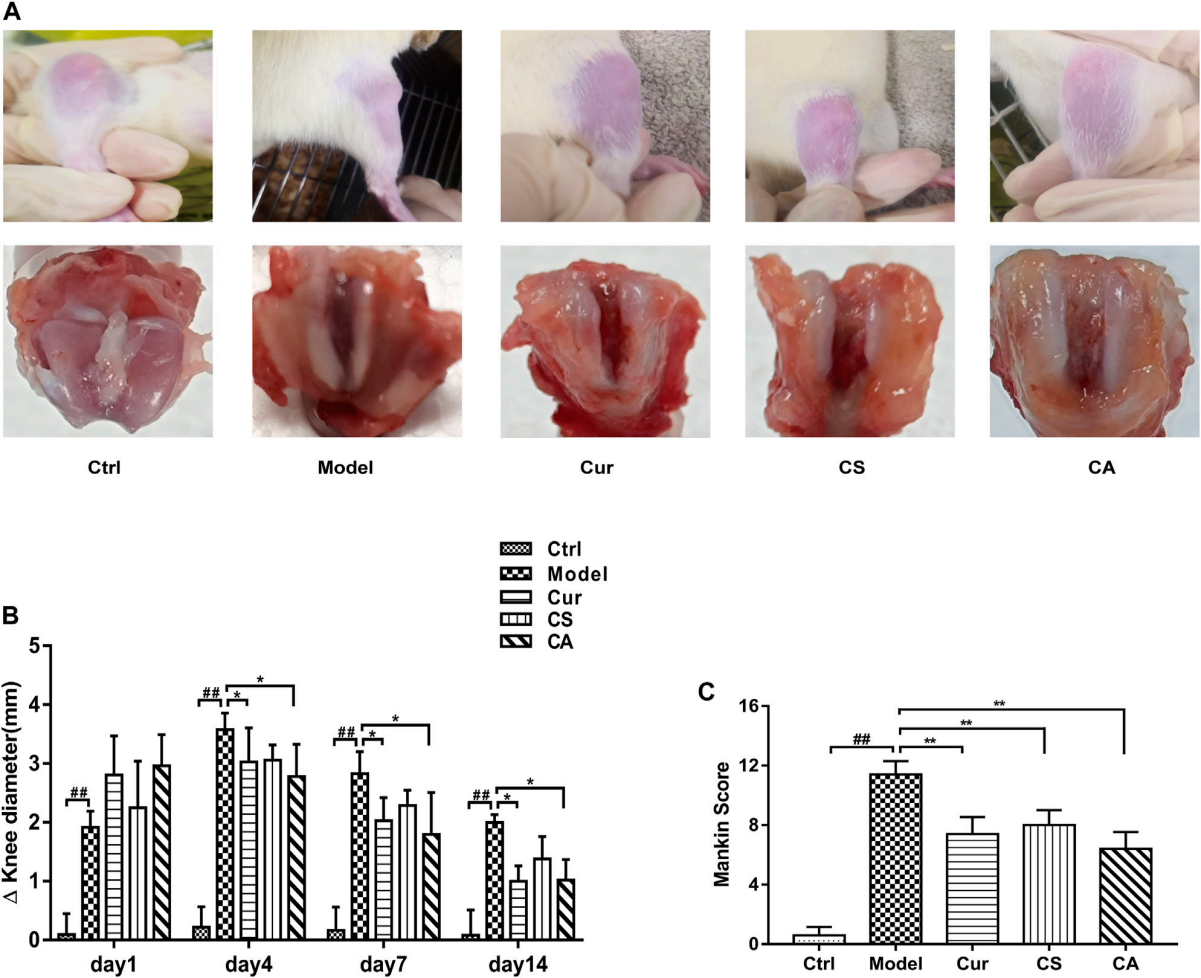
Effect of different treatments on knee osteoarthritis in MIA-induced rats. **(A)** Representative image of knee joint in each group. **(B)** Knee joint diameters of each group (*n* = 6). **(C)** Mankin scoring of each group (*n* = 5). Data are presented as mean ± SD. ^##^ *p* < 0.01 vs. Ctrl group; *p < 0.05, **p < 0.01 vs. model group.

There was no significant change in the joint diameter of the normal control group, and the knee joint could flex freely. In the MIA-induced group, however, the injured knee was unable to bend and remained inflamed and gradually recovered until the 14th day after the onset of arthritis. From the joint profile, the model group had some damage on the surface, and the blood vessels in the joint head were obviously red, and the recovery of the co-administration group (CA) approached the normal group (Figure 1A). In the co-administration (CA) and curcumin (Cur) treatment groups, the knee injury caused by MIA was significantly repaired because the diameter of the knee joint was significantly decreased, and the injured knee joint could bend and reduce edema (*p* < 0.05). (Figures 1B,C).

### CA Inhibited KOA Development in Synovial Tissues of Rats

**FIGURE 2:**
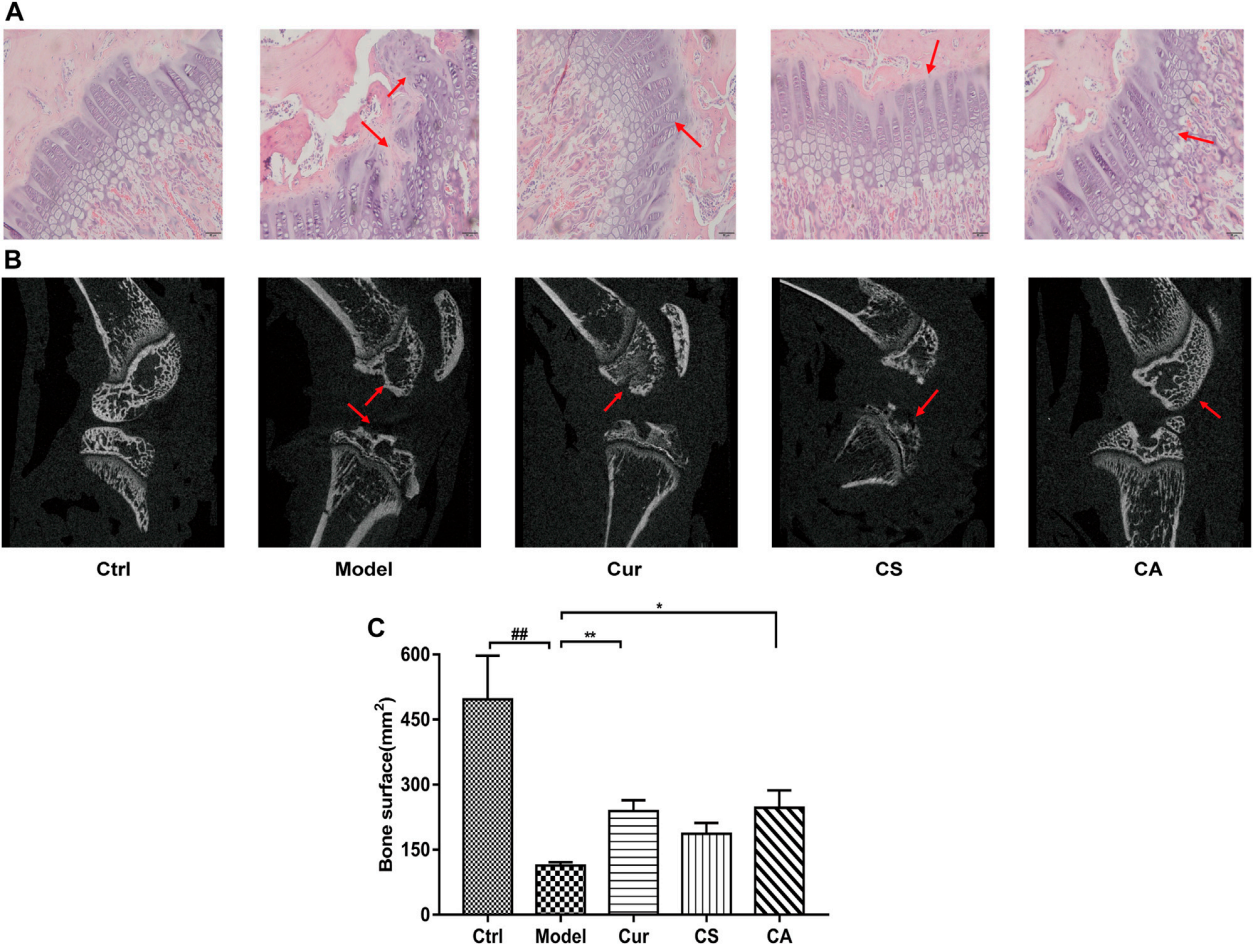
Protection of different administration groups on knee osteoarthritis in MIA-induced rats. **(A)** Representative histological staining and **(B)** CT scanning of the knee joint of different groups. Scale bar = 50 μm. **(C)** Bone surface. Data are presented as the mean ± SD (*n* = 4). ^##^ *p <* 0.01 vs. Ctrl group; *p < 0.05, **p < 0.01 vs. model group.

The outcomes of hematoxylin-eosin (H&E) staining are shown in Figure 2A. Regular cellular morphology of joint synoviocyte and neat arrangement could be observed in the normal control group. Disordered arrangement, partial necrosis and exfoliation, tissue edema, and interstitial osteoporosis of synoviocyte could be found in the KOA model group. Synoviocyte edges were slightly irregular in the Cur, CS, and CA groups with complete tissue structures.

The CT scan results of rat knee joint showed that no obvious bone changes were found in the normal group, the joint space was obvious, and no bone destruction was found. In the model group, the tide mark was broken, and the local bone hyperplasia of the knee joint, the poor articular surface integrity, and the narrowing of the joint space, indicated the formation of obvious KOA. Compared with the model group, the joint changes of the CA group tended to return to normal, the knee joint space became smaller, the cartilage recovered better, and the various conditions of KOA disease decreased (Figure 2B).

The bone surface area of each group could be obtained by analyzing the data collected from CT images. The recovery of the articular cartilage in the CA group and Cur group was improved significantly (*p* < 0.05 or *p* < 0.01) (Figure 2C).

### CA Repaired Cartilage Matrix in the Articular Cartilage Tissues of MIA-Induced KOA Rats

**FIGURE 3:**
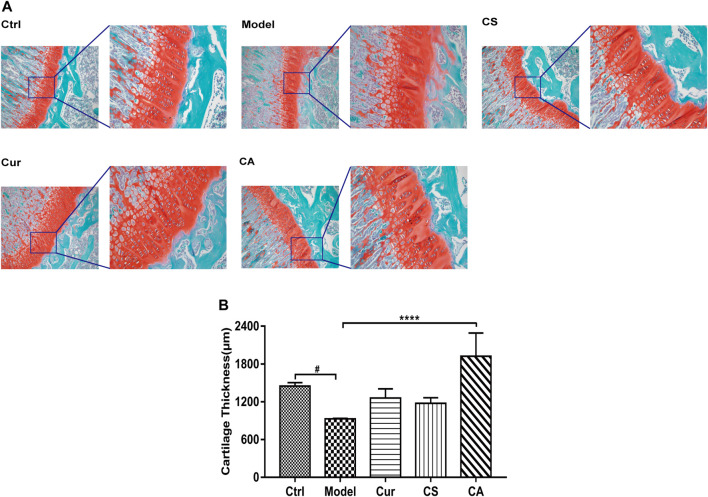
Repair of the injured cartilage in each KOA group. **(A)** Safranin fast green staining image of each group. **(B)** Cartilage thickness of each group. Data are presented as the mean ± SD (*n* = 4). ^#^ *p <* 0.05, vs. Ctrl group; ****p < 0.0001 vs. model group. Scale bar = 100 μm.

Stained knee joint sections of the control group showed that, in the tangential zone articular, surfaces of cartilage exhibited regular smooth surfaces with underlying small, flat chondrocytes. In the MIA-induced KOA group, obvious unstained knee joint sections suggested serious chondrocytes loss (Figure 3A). In the CA group, the stained knee joint showed a mostly intact tide mark and normal chondrocyte growth.

The thickness of the synovium-cartilage layer of each group was determined, and data showed that MIA induced a significant decline in the thickness of synovium when compared to the normal control group (*p <* 0.05). CA treatment could significantly increase the thickness of the synovium when compared to the saline-treated group (*p* < 0.0001).

### CA Reduced Serum Inflammation in MIA-Induced KOA Mice

**FIGURE 4:**
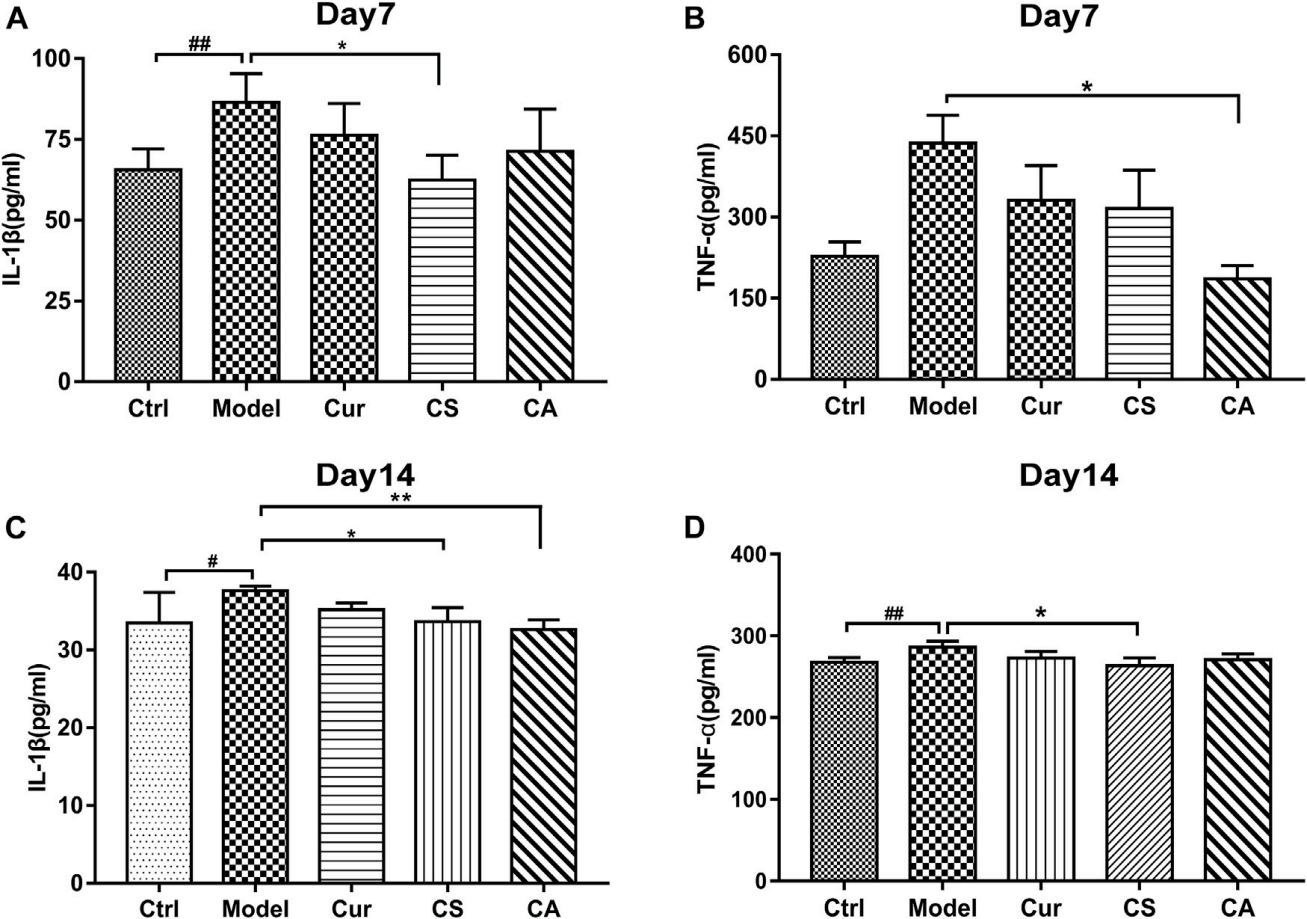
Serum IL-1β and TNF-α of MIA-induced KOA rats in each group at days 7 and 14. **(A)** Serum IL-1β at day 7 after KOA induction. **(B)** Serum TNF-α at day 7 after KOA induction. **(C)** Serum IL-1β at day 14 after KOA induction. **(D)** Serum TNF-α at day 14 after KOA induction. Data are presented as the mean ± SD (*n* = 5). ^#^ *p <* 0.05, ^##^ *p <* 0.01 vs. Ctrl group; *p < 0.05 vs. model group.

In this study, the serum levels of IL-1β and TNF-α were measured at day 7 (Figures 4A,B) and day 14 (Figures 4C,D) after KOA induction. We found that the expressions of IL-1β and TNF-α significantly changed in the MIA-induced KOA group compared with those in the normal control group. The levels of TNF-α and IL-1β were significantly downregulated by CS treatment on day 14 (*p* < 0.05). After 14 days of administration, the CA group showed a decline in elevation of serum IL-1β (*p* < 0.05).

### CA Decreased Oxidative Stress and Inflammation in Synovial Tissues of KOA Rats While Increasing Collagen II

**FIGURE 5:**
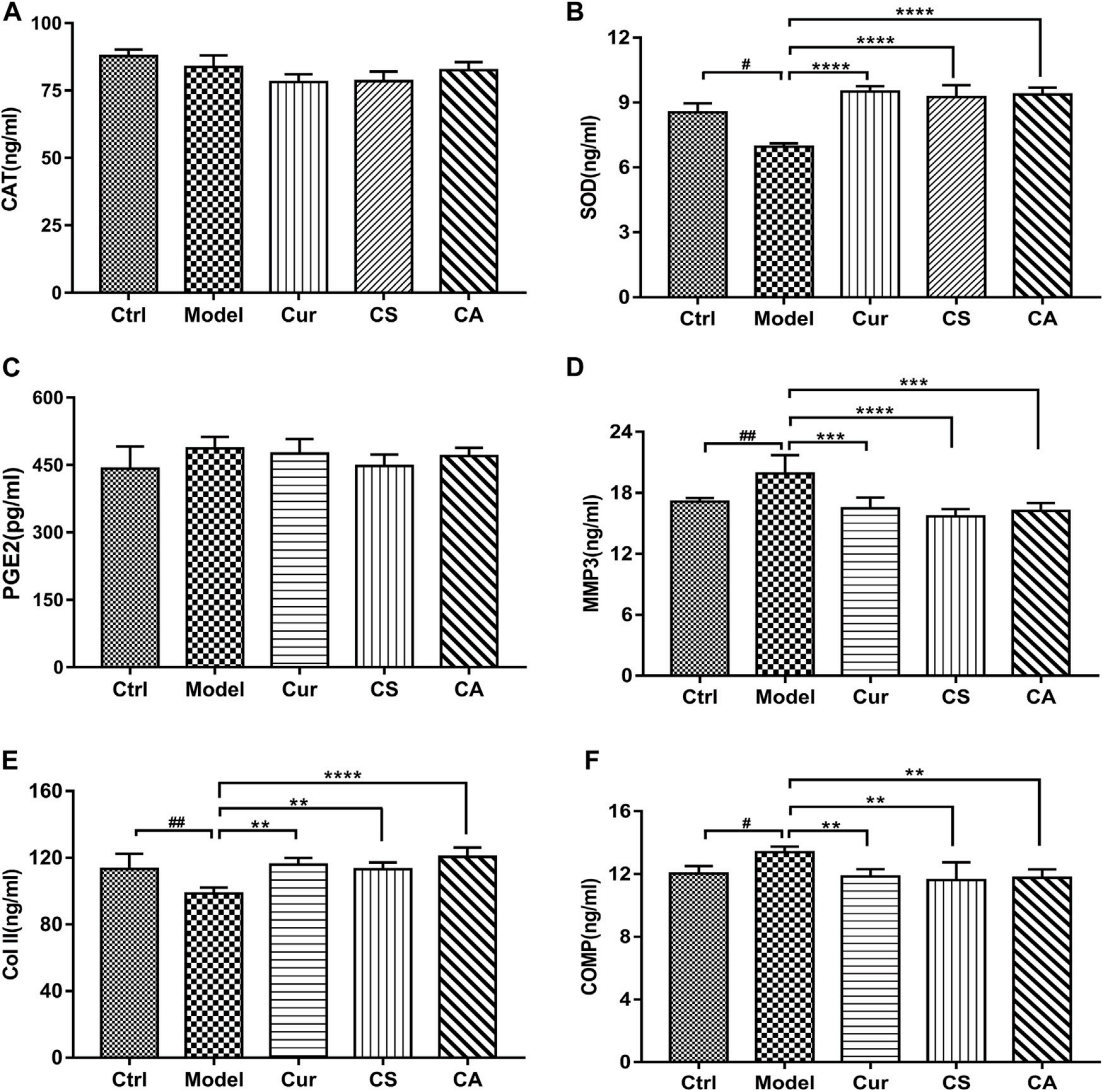
Determination of the contents of CAT, SOD, PGE2, MMP3, Col-II, and COMP in each group. **(A)** Levels of CAT in serum. **(B)** Levels of SOD in serum. **(C)** Levels of PGE2 in serum. **(D)** Levels of MMP3 in serum. **(E)** Levels of collagen II in articular cavity fluid. **(F)** Levels of COMP in articular cavity fluid. Data are presented as the mean ± SD (*n* = 5). ^#^ *p <* 0.05 vs. Ctrl group; *p < 0.05, **p < 0.01, ***p < 0.001, ****p < 0.0001 vs. model group.

The activities of SOD (Figure 5B), Col-II (Figure 5E), and COMP (Figure 5F) in serum changed significantly in the MIA-induced KOA group as compared to those in the control group (*p <* 0.05). Cur treatment could significantly increase the activities of SOD (*p <* 0.0001) and downregulate the levels of MMP3 (*p <* 0.01) and COMP (*p <* 0.05). Both CS and CA treatment could increase the activities of SOD (*p <* 0.0001) and downregulate the levels of MMP3 (*p <* 0.001) and COMP (*p <* 0.01) significantly. Administration of Cur could increase the level of collagen II (*p <* 0.01), and the CA group obviously upregulated the level of collagen II (*p <* 0.001) when compared to model groups.

### CA Inhibited the Activation of the TLR4/NF-κB Pathway in the Articular Cartilage of MIA-Induced KOA Rats

**FIGURE 6:**
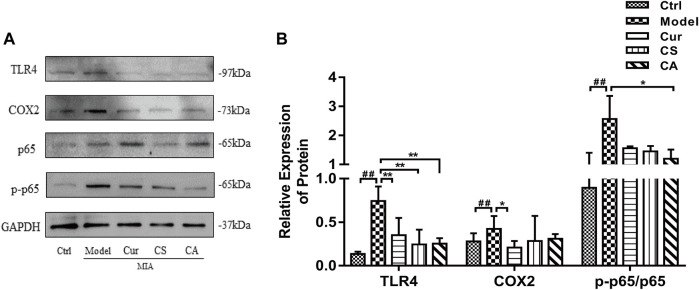
Determination of the effects of Cur, CS, and CA on the protein expression of TLR4, cox-2, NF-κB p65, and p-p65 in KOA rats. **(A)** Western blot analysis of TLR4, cox-2, NF-κB p65, and p-NF-κB p-p65 expressions in cartilage. **(B)** Relative expression of TLR4, cox-2, and p-NF-κB p-p65 divides NF-κB p65 in cartilage. Data are presented as the mean ± SD (*n* = 3). ^##^ *p* < 0.01 vs. Ctrl group; *p < 0.05 vs. model group.

The overexpression of inflammatory cytokines is related to the activation of the TLR4/NF-κB signaling pathway. Therefore, we detected the protein expressions of TLR4 and NF-κB signal pathways in the knee cartilage (Figures 6A,B). Cur, CS, and CA treatments could inhibit the expression of TLR4 (*p <* 0.01, Figure 6B). Cur treatment could also suppress the expression of cox-2 (*p <* 0.05, Figure 6B). Administration of CA downregulated the expression of p-p65 (*p <* 0.05, Figure 6B).

## Discussion

Curcumin and chondroitin sulfate, as food supplements, are considered to be effective in reducing KOA symptoms by attenuating inflammation, oxidative stress, and repairing cartilage separately. In this experiment, we demonstrated that oral co-administration of curcumin and chondroitin sulfate (CA) not only better attenuated cartilage damage but also alleviated the inflammatory response in KOA rats. Compared with the other groups, the injured knee joint was able to regain joint flexion in a shorter time, and the range of motion was better improved in the CA-treated group during KOA progression. Cur and CA had similar protective effects on knee edema, CS showed less effect, and CA had better protective effect.

According to H&E staining and safranin fast green staining of cartilage tissues, as well as the micro-CT reports, we can find that CA alleviated the cartilage degeneration and delayed the progression of osteoarthritis more obviously than Cur or CS treatment. The bone surface and the joint space, especially the thickness of the synovium, were better repaired in CA-administrated rats. These results suggest that co-administration of curcumin and chondroitin sulfate deserves to be recommended to KOA subjects.

Osteoarthritis (OA) is mainly characterized by synovial inflammation (synovitis), cartilage destruction, and bone flab formation. When OA occurs, degradation of type II collagen exceeds its synthesis, leading to an increase in serum C-terminal telopeptide of collagen type II (CTX-II). The fragments of type II collagen induced inflammation, promoted upregulation of IL-1β, and lead to apoptosis of chondrocytes (Zhou et al., 2008). IL-1β has been shown to induce cartilage matrix degradation, inhibit proteoglycan and collagen synthesis, and promote matrix metalloproteinase (MMP) production (Heraud et al., 2000; Schuerwegh et al., 2003). In addition, oxidative stress plays an important role in the physiology and pathology of KOA, and the enzymatic cellular antioxidant defense system includes various classes of enzymes, such as SOD, CAT, and GPX (Tudorachi et al., 2021). Meanwhile, the concentration of cartilage oligomeric matrix protein (COMP) in the blood may be a candidate indicator of early cartilage lesions in arthritis (Saxne and Heinegard, 1992; Saxne and Heinegard, 1995). In the present study, we tested the levels of inflammation (IL-1β, TNF-α, PGE2, MMP3, and COMP), oxidative stress (SOD and CAT), and type-II collagen after the induction of KOA. Compared to the protective effects of different administrations, data showed that the therapeutic effect of CA is mainly exerted by increasing the content of collagen II more significantly than a single treatment of curcumin or chondroitin sulfate. This finding further suggested that co-administration of curcumin and chondroitin sulfate could better repair cartilage injury in osteoarthritis rats.

TLR4, a member of the toll-like family of proteins, can recruit the MyD88 protein, which in turn triggers a signaling cascade resulting in the activation of the downstream NF-κB signal pathways. Activation of NF-κB could increase the induction of pro-inflammatory cytokines and growth factors, eventually leading to the development of knee joint injury, of which p65 is a major functional subunit in the NF-κB family (Guha and Mackman, 2001; Suryono et al., 2005). Studies suggest that toll-like receptor 4 (TLR4) expression is elevated in osteoarthritic lesions of OA patients. In addition, TLR4 is associated with inflammatory and catabolic responses through the regulation of MMPs (Murata et al., 2019). NF-κB is a nuclear transcription factor, which plays a pivotal role in KOA pathogenesis. Once activated, it can cause an inflammatory response. The abnormal activation of NF-κB results in the loss of articular chondrocytes in a state of arrested growth and is accompanied by the production of pro-metabolic mediators, such as agglutinates and matrix proteases. These enzymes induce cartilage degradation and the production of pro-inflammatory cytokines. In addition, sustained activation of NF-κB leads to the overexpression and activation of other regulatory transcription factors that contribute to the persistence of KOA disease through the regulation of inflammatory and catabolic mediators (Tudorachi et al., 2021). Curcumin supplementation can reduce inflammation in knee osteoarthritis rats by blocking the TLR4/MyD88/NF-κB signal pathway (Zhang and Zeng, 2019). In the present study, data revealed co-administration of curcumin with chondroitin sulfate could enhance the inhibitive effects of the effect of curcumin on the TLR4/NF-κB pathway in KIA rats.

Wang et al. (2020) recently explored turmeric extract in a randomized trial to treat symptoms of knee osteoarthritis and exudative synovitis and showed that turmeric extract was more effective than placebo for knee pain, which provides some support for our study, but Wang’s study cannot more directly analyze the components in cartilage and various changes in inflammatory factors. The reason we cited Wang’s article was mainly to help prove that curcumin affects cartilage changes, but the specific ingredients and reasons are not in Wang’s article; therefore, our article describes turmeric which can work together with chondroitin sulfate to improve cartilage damage and joint inflammation in knee osteoarthritis.

In summary, the strength of our experiment we found was that co-administration of curcumin supplementation and chondroitin sulfate significantly improved chondrocyte proliferation and bone surface repair partly via enhancing the secretion of type II collagen and downregulation of the TLR4/NF-κB signaling pathway in MIA-induced KOA in rats. This study provides an option of co-treatment of curcumin and chondroitin sulfate for protecting against knee osteoarthritis. We found that the CA group did not show significantly better effects than the monotherapy group on some metrics. This may be because the dose of the single treatment group is sufficient and shows good effects, and even some indicators are already close to normal levels, so it is difficult for the CA group to show better results at the same dose. However, we found that in terms of cartilage repair, the efficacy of the combined group was significantly better than that of the single group, so the results of this experiment showed that the CA group could produce better repair effects. In terms of improving inflammation through the NF-κB pathway, some indicators in the CA group could also suggest better effects.

There are still some limitations of this study that can be addressed in future studies. The focus of our research is that curcumin and chondroitin sulfate can improve osteoarthritis through the NF-κB pathway, but we only verified the changes in the combination of the protein level and the inflammatory factor level and did not verify the full aspect of the NF-κB pathway. Therefore, follow-up studies may need to be verified from the gene level of this pathway. It will provide new ideas in the screening of drugs for the prevention and treatment of KOA and a broad prospect for future therapeutic principles.

## Data Availability

The original contributions presented in the study are included in the article/Supplementary Material, further inquiries can be directed to the corresponding authors.

## Abbreviations

CA, KOA+ curcumin and chondroitin sulfate group; CAT, catalase; collagen II, type II collagen; COMP, cartilage oligomeric matrix protein; CS, KOA+ chondroitin sulfate group; Ctrl, normal control group; Cur, KOA+ curcumin group; IL-1β, interleukin-1β; KOA, knee osteoarthritis; MIA, monosodium iodoacetate; MMP-3, matrix metalloproteinase-3; model, KOA+ saline group; PGE2, prostaglandin 2; SOD, superoxide dismutase; TNF-α, tumor necrosis factor-α.
