# Supplementary figures and images for: Combined Administration of Curcumin and Chondroitin Sulfate Alleviates Cartilage Injury and Inflammation via NF-κB Pathway in Knee Osteoarthritis Rats
Source: Front Pharmacol. 2022 May 19;13:882304. doi: 10.3389/fphar.2022.882304 (PMC9161211; doi:10.3389/fphar.2022.882304)

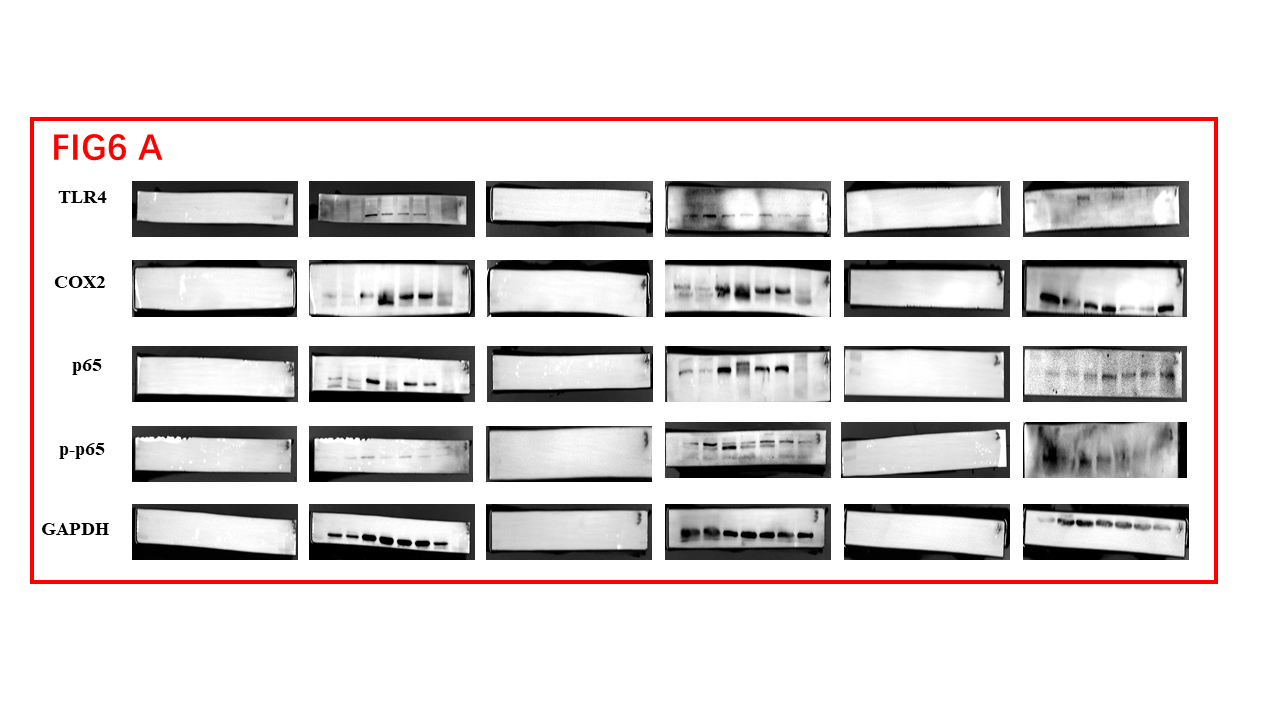

Supplement: Supplementary file 1 [file Image1.JPEG]
